# Molecular control of HIV - 1 postintegration latency: implications for therapeutic strategies

**DOI:** 10.1186/1742-4690-9-S1-I10

**Published:** 2012-05-25

**Authors:** Carine Van Lint

**Affiliations:** 1 Gosselies Campus, Charleroi, Belgium

## 

The current antiretroviral therapy HAART is effective and life-prolonging but does not eradicate HIV-1 from infected patients. A reduction of HIV-1 RNA levels in the plasma in HAART-treated individuals to less than 50 copies/ml is frequently achieved but residual viremia persists as detected by ultrasensitive assays. The sources of this persistent viremia are still not fully understood but could arise from ongoing cycles of residual viral replication and/or from the reactivation of viral expression from latently-infected cells. These latently-infected cells contain stably-integrated, transcriptionally-silent but replication-competent proviruses, thereby representing latent reservoirs of HIV-1. They are a permanent source for virus reactivation and could be responsible for the rebound of plasma viral load observed after HAART interruption.

HIV-1 transcriptional repression is crucial to the establishment and maintenance of postintegration latency. Several elements contribute to HIV-1 transcriptional repression including: 1) the site of integration and mechanisms of transcriptional interference, 2) the absence of crucial inducible host transcription factors, 3) the presence of transcriptional repressors, 4) the nucleosomal organization of the HIV-1 promoter, 5) the epigenetic control of the HIV-1 promoter (histone posttranslational modifications, such as acetylation and methylation, and DNA methylation), 6) the sequestration in an inactive form of the cellular positive transcription elongation factor b (P-TEFb), composed of cyclin-dependent kinase 9 (CDK9) and human cyclin T1, 7) the absence of the viral transactivator Tat, which promotes transcription via recruitment to the HIV-1 promoter of P-TEFb, histone-modifying enzymes and ATP-dependent chromatin-remodeling complexes required for nucleosomal disruption and transcriptional processivity. The involvement of these elements in postintegration latency depends on the status of activation and differentiation of the heterogeneous CD4^+^ T cell populations hosting the HIV-1 reservoirs.

Further understanding of the epigenetic and non-epigenetic mechanisms regulating HIV-1 latency and reactivation from latency should help devise novel strategies to eliminate latent HIV-1 infection or to restrict the latent pool to a size bearable by the host immune system.

